# Presentation of a Patient, the Subject of Calculous Formations in the Kidney, Ureter and Bladder

**Published:** 1903-12

**Authors:** Bransford Lewis

**Affiliations:** Saint Louis, Mo.; Professor of genitourinary surgery, Marion Sims-Beaumont Medical College ; genitourinary surgeon to the Rebekah Hospital, Deaconess’s Hospital, City and Female Hospitals, Mount Saint Rose Hospital, etc.


### Presentation of a Patient, the Subject of Calculous Formations in the Kidney, Ureter and Bladder.

### The Other Kidney Inactive; Removal of Stones from the Only Active Kidney by Nephrolithotomy, and from the Bladder by Means of the Operative Cystoscope of the Author.[1]

By BRANSFORD LEWIS, M. D., Saint Louis, Mo.;

Professor of genitourinary surgery, Marion Sims-Beaumont Medical College; genitourinary surgeon to the Rebekah Hospital, Deaconess's Hospital, City and Female Hospitals, Mount Saint Rose Hospital, etc.

THIS patient has been the subject of stone formation in his urinary organs to a remarkable degree; and while one kidney has been practically destroyed for some time, and the other one has been opened, cleaned and drained, and various other operations have been performed on him, he is, as you see, apparently in pretty good general condition.

---

1. Read before the Saint Louis Medical Society, October 17, 1903.

Hugh S. S., until recently a soldier in the United States Army; aged 28; nativity, Tennessee. In sailing to the Philippines in 1899, he first felt the urinary symptoms, that later became very severe. There was frequency and burning connected with urination; and after arriving at Manila three little stones passed from bladder and urethra, one of them lodging at the urethral orifice, and having to be pulled out by a surgeon there. One year later, more passed in the same way. He returned to this country, going to the City Hospital, because of further burning and pain, frequency, and the like, in urination. Dr. Nietert removed a stone from the bladder by suprapubic incision, in 1902; it was about the size of a bird's egg, and is shown herewith. One week later, the patient suddenly felt a severe pain in his right side, running down into the right testicle; it disappeared shortly afterwards, and has not recurred since,—an interesting fact, in view of the subsequent developments.

My attention was first called to him by Dr. Nietert, in April, 1903, when he was passing exceedingly foul, cloudy, purulent urine, slightly acid in reaction. We did double ureter-catheterisation at one séance, draining the urines from the two kidneys at once. In twenty minutes the right kidney sent down ten drams of urine, the left one only one and three-fourths drams. The urine from the right kidney was of fairly good, yellow color, 1008 specific gravity, cloudy from pus, and also contained some blood corpuscles, renal and ureteral epithelia, and a few hyaline casts. The urine from the left kidney was of pale, milky color, highly cloudy, specific gravity 1002, containing large quantities of pus corpuscles, renal and ureteral epithelium, and some blood corpuscles. This finding, especially when taken in consideration with the similar result of another double ureter catheterisation, done on May 5, indicated that the left kidney was doing practically no work; all of the urinary excretory work was being carried on by the right kidney. And yet this right kidney was badly afflicted—not alone with the suppurative inflammation, as indicated from the urine coming from it, but also from the fact that after each of the two ureter catheterisations, a number of small ureter stones passed from the bladder, and in each instance he could feel them pass from the right ureter into the bladder, he said. Some of these stones were caught in cheese-cloth, at the time of urination, and I present some of them to you; the remainder, probably twenty or more, were lost by the breaking of the bottle.

Although the patient felt somewhat relieved after the passing of the calculi each time, there was no clearing up of the symptoms and troublesome conditions present; the urine remained highly cloudy, purulent and foul-smelling; frequency of urination was excessive, and the patient's general condition was not improving. Believing that more calculi existed in the right kidney, I suggested that an *x*-ray photo be taken, which was carried out by Dr. John Young Brown, at the hospital; the result appearing in the print I show you here. It shows two dark spots to the right of the spinal column, one above the other. These we took to be stones.

The question of removing them, because of the existing conditions, was a serious one. We knew that the left kidney was not functionating to any practical degree: he was depending on one kidney, only, for renal excretion, and that one contained the two stones. If they remained there, it was only a question of time when it, too, would be destroyed and the patient's life would be ended. On the other hand, opening of the kidney, under the circumstances, entailed the risk of causing suppression of urine and having an immediately fatal result; but if he should go through the operation successfully, the chances of recovery would be fair. These matters were candidly told to the patient, who declared that he would rather take the chances offered by the operation than the unfortunate certainty offered by the nonoperative plan.

### NEPHROLITHOTOMY.

By courtesy of Dr. Brown and with the assistance of himself and his internes, on May 26, 1903, I cut into the right lumbar region, incised the convexity, pushed my finger down into the kidney pelvis, and felt and removed the stone, which I pass to you. Feeling in the substance of the lower pole of the kidney, I discovered a calcareous mass, not a formed stone, which was removed by scooping with the finger and washing with a stream of water. A flexible ureter-catheter was passed down the ureter, meeting with no obstruction. The major part of the kidney incision was sewed up, enough of an opening being left for drainage by the gauze packed into it. The patient recovered from the effects of the operation with surprising ease and promptness, urine appearing in the bladder on the same evening, and there was only transient rise of temperature. He was up and walking around in three weeks; and gained much weight and strength during the next two months.

Shortly after returning from my vacation, in September, the patient again called on me, complaining of irritation at the bladder neck and frequency of urination; he said he believed there was another stone in the bladder. He has had much experience in this line, and felt that he was in position to judge. On looking into the bladder, I found that he was correct; a white stone of oval shape, a half inch in thickness by three-quarters of an inch in length, lay in the *bas fond*.

LITHOLAPAXY AND REMOVAL OF FRAGMENTS THROUGH THE OPERATIVE CYSTOSCOPE.

Through my newly developed operative cystoscope, shown herewith, I introduced an alligator forceps, catching the stone and breaking off parts of it, removing them at the same time. The forceps had not been constructed for such work, however, and were not strong enough to break the stone satisfactorily; so the cystoscope was temporarily withdrawn and the stone crushed with a lithotrite; several fragments were washed out through the washing tube; when no more came, the operative cystoscope was again introduced and other fragments were detected and removed. They are shown in the bottle which I present. It was apparent that stone fragments could readily be left in a bladder after crushing and washing, especially small ones, hugged in the folds of the mucous membrane; and that the operative cystoscope would offer much advantage in detecting and removing them. However, there was one, larger, fragment noted in the bladder, which I thought would be interesting to Dr. Brown, so I left it there, for the time being. The City Hospital Medical Society was scheduled to meet at the City Hospital about this time, and we both thought that a demonstration of the stone within the bladder would be of interest to its members; which was made on the evening of September 5th; after which the remaining fragment was further broken and removed through the operative cystoscope, which cleared the bladder of foreign material. The patient has been relieved subjectively since then; although his urine is not yet clear and there are evidences of subacute inflammation in parts of the urinary tract, the pelvis and bladder, especially, for which we expect to give him regular medicated irrigations,—the pelvis being washed directly by means of the ureter catheter, introduced through the catheterising cystoscope.

With the exception of the lithotomy done by Dr. Nietert, and the nephrolithotomy, performed later, all of these various procedures have been carried out with local anesthesia, secured with cocaine. If general anesthesia had been necessary each time, it is probable that material damage would have been done to this sole remaining (so far as functionation is concerned) kidney. The patient is now passing better urine than he has passed at any time since I have been acquainted with him. He is also stronger and in better general condition. He has gained several pounds in weight.

It will be noted that this patient has contributed largely as a subject for developing comparatively untrodden paths of surgical work.[1] The removal of calculi from the ureter by noncutting methods, and the breaking and removal of stones from the bladder through a cystoscope, are methods that, to say the least, have not been undertaken with any degree of assurance, if they have been carried out as a planned attack; and the successful removal of calculi from the only working kidney, is a record that should be of service in guiding other surgeons in a similar situation.

To show that the attempt to remove stones from the ureter, by uretercystoscopic methods, has been attended with an encouraging amount of success during the past year or more, I show you a number of calculi that have been obtained in that way. I shall report the cases together at some later date. Suffice it to say now that, so far, I have had four cases in which intraureter manipulations of various sorts have been followed by the escape of calculi that had been impacted a greater or less length of time in ureters.

The methods I have been working on are as yet immature, the instruments in process of development with plenty of room for improvement, and the problem undertaken a large one; nevertheless, sufficient success has been attained to let us hope that much may be done in the direction aimed at. If it is ever secured, a material step in conservative surgery will be attained. The removal, by a cutting operation, of stones impacted in the ureter, especially in the middle or lower portion, is an operation that is very inviting, even under the best of conditions; and it must be remembered that a large portion of such cases are already the subject of renal infection and degeneration, with heart complications not unusual. Opening a ureter through the peritoneum is precarious, to say the least; and in a corpulent person, to follow the ureter down from the kidney outside of the peritoneum by prolonged incision, or to go in directly through an incision toward the iliac fossa, seeking the ureter in the depths of the wound, are measures attended with much difficulty or even failure. So that, to repeat, there are abundant and emphatic reasons why the ability to remove ureter stones without a cutting operation would be a long step in conservative surgery.

We will hope it may be accomplished.

627 Century Building.

---

1. Hugh Young, in *American Medicine*, August 9, 1902, details the removal by him, with the aid of a catheterising cystoscope, of a stone located in the orifice of a ureter, in a male patient; and Howard Kelly, in the *Journal of American Medical Association*, March 3, 1900, reports the passage of a calculus from the ureter of a woman, after his dilating the contracted orifice of the ureter by means of sounds. These are the only instances, so far as I know, in which ureter stones were removed with the aid of the cystoscope.

